# Plasma microbial cell-free DNA load is associated with mortality in patients with COVID-19

**DOI:** 10.1186/s12931-021-01623-0

**Published:** 2021-01-20

**Authors:** Georgios D. Kitsios, William Bain, Nameer Al-Yousif, Radha Duttagupta, Asim A. Ahmed, Bryan J. McVerry, Alison Morris

**Affiliations:** 1 Division of Pulmonary, Allergy and Critical Care Medicine, Acute Lung Injury Center of Excellence, University of Pittsburgh Medical Center, UPMC Montefiore Hospital, NW 628, 3459 Fifth Avenue, Pittsburgh, PA 15213 USA; 2 Veterans Affairs Pittsburgh Healthcare System, Pittsburgh, PA USA; 3 Department of Medicine, University of Pittsburgh Medical Center Mercy, Pittsburgh, PA USA; 4 Karius Inc, Redwood City, CA USA

## Introduction

Severe COVID-19 pneumonia requiring intensive care unit (ICU) support can be complicated by secondary bacterial or fungal infections. The incidence and impact of secondary pneumonias in COVID-19 are not well-defined because clinical distinction from isolated SARS-CoV-2 infection is challenging and diagnostic practices have been highly variable [1]. Early administration of empiric antibiotics limits the sensitivity of subsequent microbiologic studies, whereas standard invasive workup with bronchoscopy is often avoided due to the risks of healthcare personnel exposure to aerosolized SARS-CoV-2 [2]. To overcome such limitations and comprehensively identify secondary pneumonias in COVID-19, we performed microbial cell-free DNA (mcfDNA) metagenomic sequencing (mcfDNA-Seq) in plasma samples in addition to conventional microbiologic workup.

## Methods

We enrolled 15 critically-ill patients with COVID-19 (confirmed by nasopharyngeal qPCR for SARS-CoV-2) in a prospective ICU cohort study [3]. Following informed consent, we obtained plasma samples for conducting mcfDNA-Seq with the Karius Test (Karius, Inc. Redwood City, CA)[4]. We evaluated detection of mcfDNA in the context of clinical diagnoses and prescribed antimicrobial therapies by the treating physicians, and examined for associations with clinical outcomes.

## Results

**Fig. 1:**
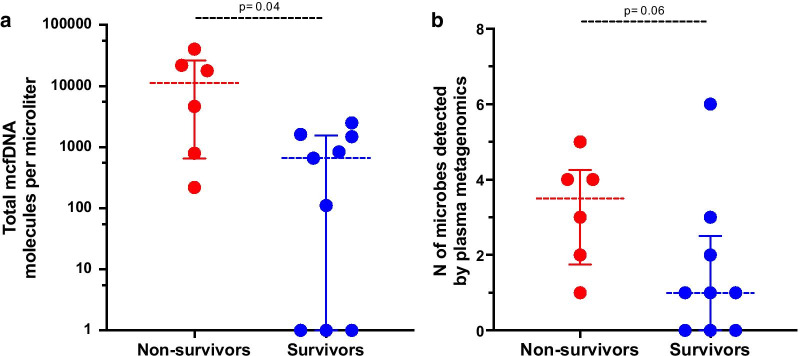
Non-survivors of severe COVID-19 infection had higher microbial cell-free DNA molecules per microliter of plasma by metagenomic sequencing compared to survivors (median [interquartile range]: 11,125 [650–26,436] vs. 661 [1], Wilcoxon test p-value = 0.04) and a trend for higher number of identified microbes per sample (3.5 [1.8–4.3] vs. 1.0 [0–2.5], Wilcoxon test p-value = 0.06)

Of 15 patients analyzed (median age 63, 53% females, 73% mechanically-ventilated), six (40%) died within 30 days from enrollment. Samples were obtained at a median (interquartile range-IQR) of 10 (4–12) days from COVID-19 symptoms onset, and each sample contained a median of 837 (111–4638) total mcfDNA molecules per microliter (MPMs) and 2 (1–4) identified organisms. Of the total 92,791 MPMs reported across 15 samples, 90% belonged to typical pathogenic bacteria (e.g. *E.coli* and *K. Pneumoniae*), with the remainder MPMs aligned to commensal bacteria (5%, e.g. oral *Streptococcus* species), fungi (4%, *Candida* species) and DNA viruses (1%). Compared to survivors, non-survivors had higher total mcfDNA (p = 0.04), higher pathogenic bacteria MPMs (p = 0.02) and a trend for a higher number of identified organisms per sample (p = 0.06). (Fig. 1).

**Fig. 2:**
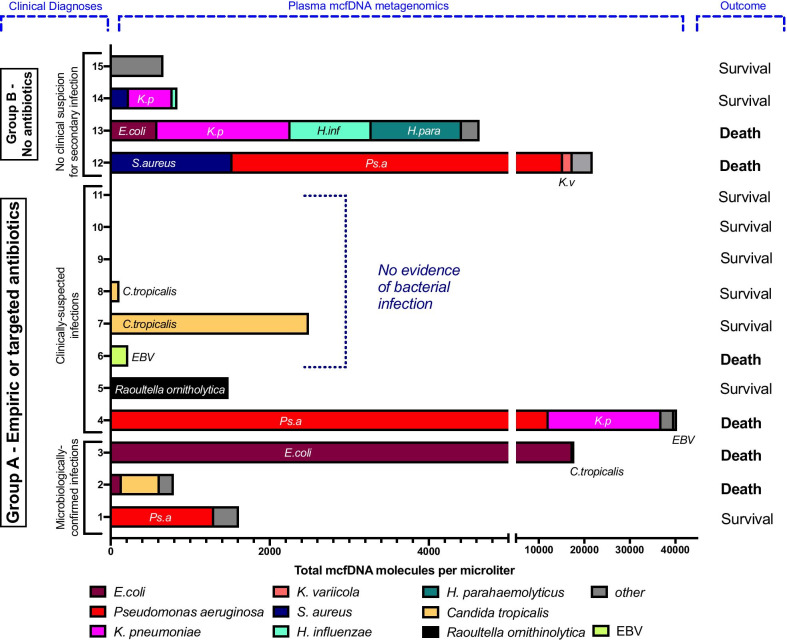
Case-based analysis of 15 critically ill patients with COVID-19 with depicted clinical diagnoses, plasma microbial cell-free DNA metagenomics and survival outcomes. The Y-axis margin indicates two groups of clinical diagnoses: Group A includes 11 patients who received antibiotics for either microbiologically-confirmed (n = 3) or clinically-suspected infections despite negative microbiologic workup (n = 8), whereas Group B includes four patients with low clinical suspicion for secondary infection and no antibiotic therapies at time of sampling. The Y-axis ticks denote each patient sample and the x-height of each stacked bar represents the number of microbial cell-free DNA molecules per plasma microliter (MPMs) by metagenomic sequencing, with different colors for the top 10 microbes by ranked abundance. The “other” category (shown in grey) represents the sum of lower abundance taxa of commensal origin. Five out of 11 subjects of Group A (45%, Subjects 1–5) had high MPM signal for probable respiratory pathogens, whereas in the remaining 6/11 subjects there was no evidence of co-infecting bacterial pathogens. Subject 7 was clinically-diagnosed with culture-negative sepsis and treated with prolonged course of empiric broad-spectrum antibiotics while on extracorporeal membrane oxygenation support for refractory hypoxemic respiratory failure from COVID-19; the high mcfDNA signal for *C.tropicalis* (2,490 MPMs) is concerning for undiagnosed invasive Candidiasis, corroborated by persistent growth of yeast organisms (not further speciated) from clinical bronchoalveolar lavage samples obtained on days 5, 9 and 14 after the research sample acquisition. Two out of four patients of Group B (subjects 12 and 13) who did not survive and had not received empiric antimicrobials were found to have high mcfDNA signal (> 4000 total MPMs) of probable respiratory pathogens, indicative of undiagnosed (and untreated) secondary infections

Secondary pneumonia was clinically suspected or diagnosed by the treating physicians in 11/15 (73%) patients (Group A, Fig. 2), with microbiologic confirmation by positive respiratory cultures in 3/11 subjects (27%); these three patients had high plasma mcfDNA MPMs for common bacterial pathogens, such as *E.coli* and *Ps. aeruginosa*. Among the remaining eight patients with clinically-suspected infections and empiric antibiotic treatments, high mcfDNA MPMs of probable bacterial pathogens were detected in 2/8 patients (co-infecting *Ps. aeruginosa* and *K. Pneumoniae*; *Raoultella ornithinolytica*, respectively). In the additional six patients, no evidence of co-infecting bacterial pathogens was present, whereas in one patient (subject 7, Fig. 2) there was high signal for *Candida tropicalis* (2,490 MPMs) concerning for undiagnosed invasive Candidiasis.

We detected respiratory pathogen MPMs (*S. aureus*, *Ps. aeruginosa* and *K. Pneumoniae*) in 3/4 subjects with low suspicion for secondary infection (Group B, Fig. 2). In these patients, no respiratory specimen cultures were obtained, and antibiotics had not been initiated or had been discontinued based on negative blood cultures by the time of research sampling. Notably, two of these individuals experienced sustained vasodilatory shock and died from multiorgan dysfunction attributed to isolated SARS-CoV-2 infection.

## Discussion

McfDNA-Seq in patients with COVID-19 indicates a higher incidence of probable secondary infections than previously recognized. Despite our small sample size, the significant association between mcfDNA and 30-day mortality suggests that COVID-19 severity may be influenced by circulating bacterial fragments, either from secondary pneumonias or from possible translocation of colonizing microbiota along the disrupted alveolar/epithelial surface of lungs injured by COVID-19 [5]. Integration of mcfDNA detection with clinical data demonstrates opportunity for antibiotic stewardship in patients with suspected infection. On the other hand, the signal for undiagnosed and untreated secondary infections should serve as a call for vigilance and thorough diagnostic workup in patients with severe COVID-19 [6].

## Data Availability

All de-identified data are available from the authors upon request.
